# Overexpression of the human cytomegalovirus UL111A is correlated with favorable survival of patients with gastric cancer and changes T-cell infiltration and suppresses carcinogenesis

**DOI:** 10.1007/s00432-019-03092-x

**Published:** 2020-02-05

**Authors:** Xin Liu, Kangming Lin, Xielin Huang, Wangkai Xie, Dan Xiang, Ning Ding, Changyuan Hu, Xian Shen, Xiangyang Xue, Yingpeng Huang

**Affiliations:** 1 Department of General Surgery, The Second Affiliated Hospital and Yuying Children’s Hospital of Wenzhou Medical University, Wenzhou, 325035 China; 2 Department of Microbiology and Immunology, Institute of Molecular Virology and Immunology, Institute of Tropical Medicine, School of Basic Medical Sciences, Wenzhou Medical University, Wenzhou, 325006 China; 3 Department of Gastrointestinal Surgery, The First Affiliated Hospital, Wenzhou Medical University, Wenzhou, China

**Keywords:** Gastric cancer, Human cytomegalovirus, Infiltrating immune cells, Prognosis, UL111A gene

## Abstract

**Purpose:**

We previously found that human cytomegalovirus (HCMV) infection is associated with gastric cancer (GC) development. UL111A plays a role during HCMV productive or latent infection. However, UL111A expression profiles in GC tissues and their relationship with this disease are unknown.

**Methods:**

PCR and nested RT-PCR were performed to verify UL111A expression in 71 GC tissues and its transcripts in 16 UL111A-positive GC samples. UL111A expression levels in GC patients were evaluated by immunohistochemistry on a tissue microarray for 620 GC patients. The correlations among UL111A expression levels, clinicopathological characteristics, and prognosis were analyzed. Further, the effects of overexpression of latency-associated viral interleukin-10 (LAcmvIL-10) and cmvIL-10 on GC cell proliferation, colony formation, migration, and invasion were assessed.

**Results:**

The UL111A detection rate in GC tissues was 32.4% (23/71) and that of its mRNA expression was 68.75% (11/16). High expression of UL111A was also related to better overall and disease-free survival in GC patients. GC patients with TNM II/III stage expressing higher UL111A levels might benefit from adjuvant chemotherapy (ACT) after surgery. Moreover, high UL111A expression was also associated with increased CD4+ , CD8+ T-lymphocyte and Foxp3+ T-cell infiltration. In vitro assays further demonstrated that LAcmvIL-10 and cmvIL-10 overexpression inhibits GC cell line proliferation, colony formation, migration, and invasion.

**Conclusions:**

High UL111A expression changes the number of infiltrating T cells and is associated with favorable survival. Therefore, UL111A could be used as an independent prognostic biomarker and might be a potential therapeutic target for GC.

**Electronic supplementary material:**

The online version of this article (10.1007/s00432-019-03092-x) contains supplementary material, which is available to authorized users.

## Introduction

Gastric cancer (GC) remains an important type of cancer worldwide; more than 1,000,000 new cases were reported in 2018, with an estimated 783,000 deaths, making GC the fifth most frequently diagnosed cancer and the third leading cause of cancer-related deaths worldwide (Bray et al. 2018). Adjuvant chemotherapy after curative resection for GC has been verified to have survival benefits, but unfortunately, disease prognosis remains poor (Mari et al. 2000; Cunningham et al. 2006). The 5-year overall survival (OS) for GC is generally 25–30% (Allemani et al. 2015). Thus, gaining a better understanding of tumorigenesis and developing new diagnostic and therapeutic strategies for GC are still urgently needed to improve clinical prognosis for these patients.

Previous studies have shown that human cytomegalovirus (HCMV) is related to various cancers such as glioblastoma, medulloblastoma, colorectal cancer, breast cancer, prostate cancer, and lymphoma(Cobbs et al. 2002; Baryawno et al. 2011; Harkins et al. 2002, 2010; Samanta et al. 2003; Mehravaran et al. 2017). However, the role of HCMV infection in tumors is still controversial (Herbein 2018). On one hand, HCMV infection can promote tumor development (Mossman et al. 2013; Teo et al. 2017), whereas, on the other hand, some reports also indicated that HCMV infection can inhibit the transformation process in cancer cells (Oberstein and Shenk 2017; Kumar et al. 2016). Our group previously confirmed that HCMV infection is associated with the development of GC (Zhang et al. 2017; Jin et al. 2014), but the mechanism underlying its effect on the occurrence and development of GC is still not clear.

HCMV belongs to the Betaherpesvirinae subfamily and contains a double-stranded DNA genome, 236 kbp in size (Dioverti and Razonable 2016). The *UL111A* gene was first identified in the complete DNA sequence of AD169 (Chee et al. 1989) and is the only gene in the HCMV genome that encodes a cellular cytokine homolog (McSharry et al. 2012). The cmvIL-10 transcript was originally discovered by two research groups, expressed during the productive phase of infection in MRC-5 cells infected with the Towne strain and HEL299 cells infected with the AD169 strain (Kotenko et al. 2000; Lockridge et al. 2000). The LAcmvIL-10 transcript was initially identified during latent HCMV infection in human granulocyte–macrophage progenitors (GMPs) infected with the Toledo strain (Jenkins et al. 2004). Subsequent analysis revealed that the LAcmvIL-10 transcript is also expressed in productively infected human foreskin fibroblasts (HFFs) (Jenkins et al. 2008a, b). In addition to cmvIL-10 and LAcmvIL-10 transcripts, five other transcripts have also been reported in AD169-infected MRC-5 cells, but their biological function was not reported (Yi-Ling et al. 2008). However, to date, there has been no research regarding the function of UL111A in GC. Therefore, extensive work on the *UL111A* gene is essential.

In this study, we investigated the expression profiles of *UL111A* in GC tissues. We also investigated the clinical significance of the UL111A protein in GC patients. The effects of LAcmvIL-10 and cmvIL-10 on GC cell growth and metastasis were also assessed by overexpression experiments in vitro. This study thus provides a detailed understanding of *UL111A* and its functions as a novel therapeutic target for GC.

## Materials and methods

### Patients and specimens

Seventy-one patients with GC diagnosed by postoperative pathology were included in the study. These patients were treated surgically in the Second Affiliated Hospital of Wenzhou Medical University (Zhejiang Province, China). Paired specimens of GC tissues and the corresponding adjacent normal gastric tissues were obtained from these patients and stored in RNAlater Stabilization Solution (Invitrogen, Carlsbad, CA, USA). None of the patients were treated with pre-operative radiation or chemotherapy. The histological types of GC were classified based on the Lauren classification. The TNM staging of GC was according to the AJCC/UICC Classification for Carcinoma of the Stomach (8th edition). Each patient provided informed written consent and the study was performed after the approval of the Human Research Ethics Committee at the Second Affiliated Hospital of Wenzhou Medical University.

### Cell culture

The AGS human GC cell line was obtained from the American Type Culture Collection (ATCC, Manassas, VA, USA). Human GC cell lines BGC-823 and SGC-7901 and primary HFFs were obtained from the Type Culture Collection of Chinese Academy of Sciences (Shanghai, China). AGS cells were maintained in 1640 medium (Gibco, Carlsbad, CA, USA), and BGC-823, SGC-7901, and HFF cells were maintained in Dulbecco’s modified Eagle’s medium (Gibco, Carlsbad, CA, USA) supplemented with 10% fetal bovine serum (Gibco, Carlsbad, CA, USA) and (1 ×) TransMypre (TransGen, Beijing, China) at 37 °C in a humidified 5% CO_2_ atmosphere. HFFs were used to amplify AD169 virus and Merlin virus (ATCC, VA, USA).

### Construction of recombinant LAcmvIL-10 and cmvIL-10 plasmid, cell transfection, and western blot analysis

The full-length sequence of LAcmvIL-10 and cmvIL-10 from GC tissues, tagged with an HA tag, was subcloned into the pcDNA3.1(+) vector by ligating it into the BamHI/EcoRI sites. The plasmid or parental vectors pcDNA3.1(+) were transfected into AGS, BGC-823, and SGC-7901 GC cells using Lipofectamine 2000 (Thermo Fisher Scientific, IL, USA) according to the manufacturer’s protocol. The cells were lysed using RIPA Lysis Buffer (Beyotime, China) supplemented with a protease inhibitor cocktail for mammalian cell and tissue extracts (Beyotime). The concentrations of proteins were determined using an Enhanced BCA Protein Assay Kit (Beyotime), and 20 μg of proteins was separated on a 15% SDS-PAGE gel. Then, proteins were transferred to Immun-Blot PVDF Membranes with a 0.2 µm pore size (Bio-Rad, USA). After blocking with 5% skim milk for 1 h at room temperature, the membranes were incubated with primary antibodies overnight at 4 °C. The next day, the membranes were washed three times with TBST for 10 min each and then incubated with an HRP-linked secondary antibody against the corresponding species for 1 h at room temperature. GAPDH was used as an endogenous control. The expression of LAcmvIL-10 and cmvIL-10 in these transfected cells was confirmed with a polyclonal antibody against viral HCMV IL-10 (1:2000 dilution; AF117; R&D) or monoclonal antibody against the HA-Tag (1:1000 dilution; #7074; Cell Signaling Technology), respectively.

### PCR and nested RT-PCR analysis

Total genomic DNA from 71 paired gastric tumors and adjacent normal tissues was extracted using a TIANamp Genomic DNA kit (Tiangen Biotech Co., Ltd., Beijing, China) according to the manufacturer’s protocol. Total RNA was isolated from GC tissues using TRIzol Reagent (Invitrogen Life Technologies, Carlsbad, CA, USA) according to the manufacturer’s instructions, followed by cDNA synthesis using the ReverTra Ace^®^ qPCR RT Master Mix (TOYOBO, Tokyo, Japan). The amplification of DNA or reverse-transcribed cDNA was performed by nested PCR using the 2 × Phanta^®^ Max Master Mix (Vazyme Biotech Co., Ltd., Nanjing, China) or TransStart TopTap DNA polymerase Kit (TransGen biotech, #AP151) according to the manufacturer’s protocols. The *UL111A* gene-specific primer sequences used for PCR are listed in Table S2. The PCR products were visualized on a 1% or 2% agarose gel stained with GelRed (Solarbio, Beijing, china).

### In vitro tumor cell proliferation, colony formation, migration, and invasion assays

For proliferation assays, GC cells were plated in 96-well plates at a density of 5 × 10^3^ cells/well for 24 h, then, they were transfected with pcDNA3.1-LAcmvIL-10, pcDNA3.1-cmvIL-10, or control pcDNA3.1(+). Cell viability at 24, 48, and 72 h after transfection was assayed using Cell Counting Kit-8 (Dojindo, Kumamoto, Japan) and the absorbance at 450 nm was measured. For colony formation, transfected cells were plated in 6-well plates at a density of 5.0 × 10^2^ cells/well. The resulting colonies were fixed with 4% paraformaldehyde and stained with 0.2% crystal violet for counting after approximately 2 weeks of culture. Tumor cell migration and invasion were analyzed in 24-well Boyden chambers with 8 μm pore size polycarbonate membranes (Corning). For invasion assays, the membranes were coated with approximately 50 μg of Matrigel (Corning). AGS, BGC-823, and SGC-7901 cells were analyzed in the migration and invasion assays. For this, 1 × 10^5^ cells for migration and 2 × 10^5^ cells for invasion were resuspended in 200 μl serum-free 1640 or DMEM at 24 h post-transfection and were added to the upper compartments of the chambers; the lower compartments were filled with 600 μl of 1640 or DMEM with 10% FBS. After 20 h incubation for migration assays and 24 h incubation for invasion assays, the cells remaining on the upper surfaces of the membrane were removed. The cells on the lower surfaces of the membrane were fixed and stained with crystal violet. Photographs of five randomly selected fields were captured and the cells were counted with Image-Pro Plus software.

### Immunohistochemical analysis

Immunohistochemistry (IHC) on the tissue microarray (TMA) was performed manually. We performed IHC on the TMA from The Second Affiliated Hospital of Wenzhou Medical University. The TMA was constructed as described previously (Xu et al. 2017). Samples were dewaxed in xylene and rehydrated through a series of graded alcohols. Then, they were immersed in distilled water. Next, antigen retrieval was carried out using a high-pressure cooker for approximately 20 min in citrate antigen retrieval buffer (Zhongshan Golden Bridge Biotechnology, Beijing, China). Endogenous peroxidases were blocked with 0.3% hydrogen peroxidase for 10 min, followed by incubation in 5% normal donkey serum (Solarbio, Beijing, china) for 30 min to prevent nonspecific antigen binding. The sections were then incubated with an anti-Viral HCMV IL-10 Polyclonal Goat antibody (1:50; AF117; R&D) in a humidified chamber at room temperature for 2 h. After washing three times with PBST, the sections were incubated with anti-goat secondary antibody (1:500; ab6885; Abcam) for 30 min. The sections were developed in 3, 3-diaminobenzidine and were counterstained with hematoxylin. Next, the sections were dehydrated using an alcohol gradient and sealed with neutral gum.

The percentages of tumor cells that stained positive for UL111A were analyzed independently by two researchers. Positive staining for UL111A occurred in the cytoplasm and when > 50% of the tumor cells showed immunohistochemical reactivity for UL111A, samples were considered positive. The positive samples were then further subdivided into weakly positive, moderately positive, and strongly positive based on the intensity of staining. Finally, we defined the negative and weakly positive groups as the low expression group and the moderate and strongly positive groups as the high expression group.

### Statistical analysis

All statistical analyses were two-sided and were performed with SPSS version 21.0 for Windows (SPSS, Chicago, IL, USA). Categorical variables were compared by a Chi square test and continuous variables were compared by a Student’s *t* test. For survival analysis, survival curves were calculated based on the Kaplan–Meier method and compared by log-rank tests. The prognostic potentials of the parameters were analyzed by univariate and multivariate Cox proportional hazards models with stepwise forward selection. The hazard ratio (HR) and 95% confidence intervals (CI) were estimated. A Mann–Whitney *U* test was performed to compare the expression levels of UL111A with different infiltrating T cells in TMAs. A *p* value < 0.05 was considered statistically significant.

## Results

### Expression and transcript characteristics of UL111A in GC tissues

**Fig. 1:**
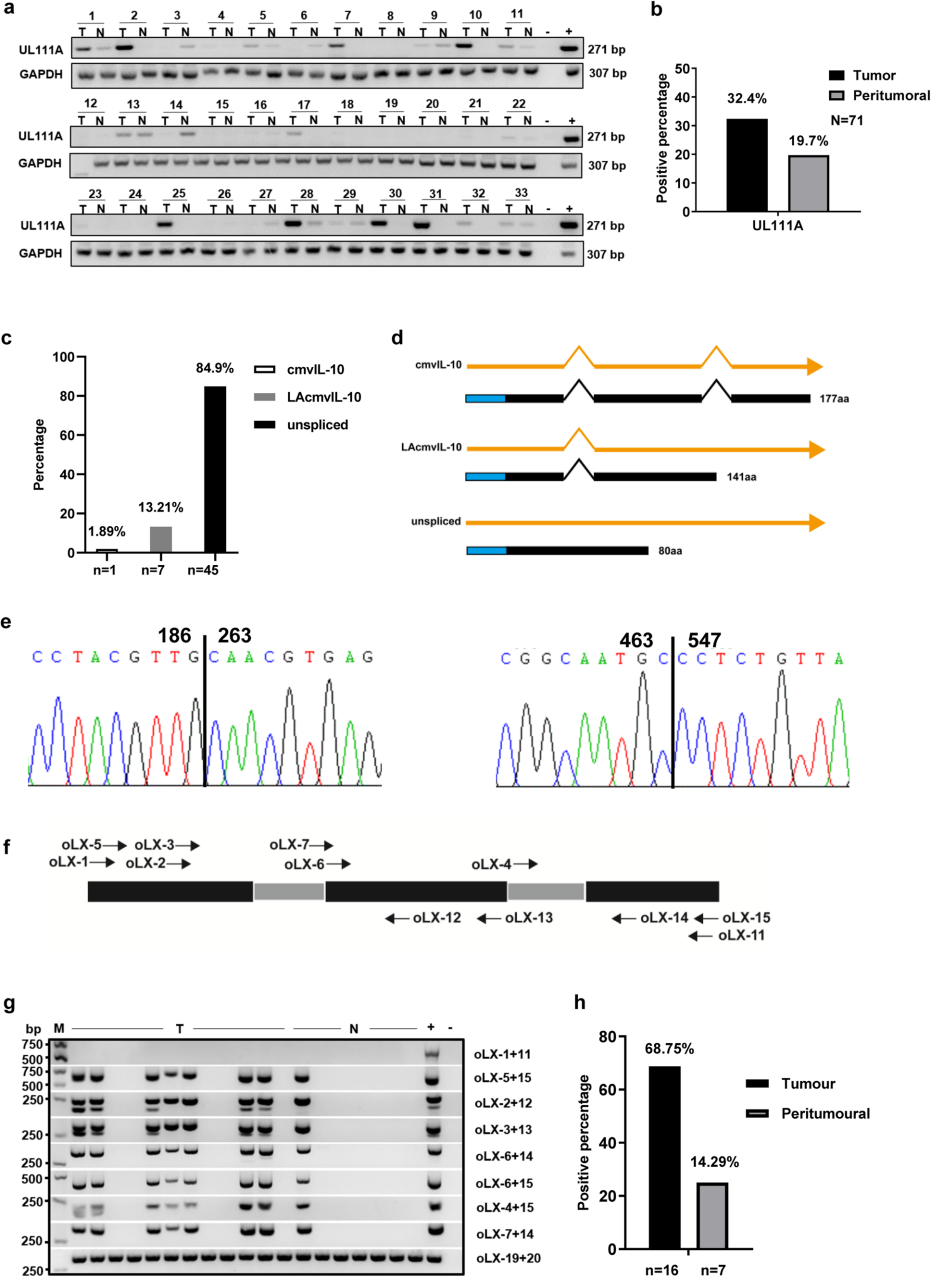
Detection of *UL111A* gene and its transcripts in gastric cancer tissues. **a** The positive percentage of the HCMV *UL111A* gene in GC tissues and peritumoral tissues using PCR. **b** Numbers represent the codes of specific patients, “T” indicates tumor and “N” indicates peritumoral, “−” indicates negative control and “+” indicates positive control, genomic DNA from an HCMV strain AD169. **c** Proportion of different transcripts of the *UL111A* gene in clones. **d** The two transcript patterns (cmvIL-10 and LAcmvIL-10) of the *UL111A* gene as well as an unspliced pattern. The blue rectangle represents the signal peptide and the black rectangle represents the encoded protein. **e** The chromatographs show the LAcmvIL-10 and cmvIL-10 sequences with nucleotide positions at the identified splice junction. **f** Location of different primers of the UL111A sequence used for nested RT-PCR analysis. **g** Nested RT-PCR detection of intron retention and abundance of unspliced UL111A on total RNA isolated from GC and peritumoral tissues. GAPDH served as an internal reference control. “−” indicates negative control and “+” indicates positive control, which is the cDNA of HFF cells infected with AD169 virus. **h** Positive percentage of *UL111A* transcripts in GC tumor and peritumoral tissues

To evaluate the potential role of UL111A in GC tissues, we analyzed the percent positivity using the oLX2 + 12 primers in tumor and peritumoral (at least 5 cm from the tumor site) tissues samples of various stages. As shown in Fig. 1a and b, the positive rate of *UL111A* detection in GC tissues was 32.4% (23/71), but was only 19.7% (14/71) in matched peritumoral tissues. To further detect the transcript patterns of *UL111A* in GC tissues, total RNA was isolated from GC tissues and analyzed by RT-PCR using the *UL111A*-specific full-length primer oLX1 + 11. Following gel purification, cloning, and sequencing of a product of ~ 690 bp (supplementary Fig. 1), we identified three transcripts, namely LAcmvIL-10, cmvIL-10, and an unspliced pattern. 7 of 45 clones harbored LAcmvIL-10, representing a single splice event, whereas 1 of 45 clones harbored cmvIL-10, which was spliced twice. In addition, the remaining clones showed unspliced patterns (Fig. 1c and supplementary Table 1). The patterns of nucleotides and proteins are shown in Fig. 1d. Furthermore, to explore the exon–exon splice junctions of these two transcripts, we analyzed the cloned sequences, and the results are shown in Fig. 1e. Next, to confirm the transcripts that we detected previously, we used *UL111A*-specific primers (Fig. 1f and supplementary Table 2) and nested RT-PCR (Fig. 1g). The diagram based on the oLX2 + 12 primers shows an upper band (271 bp), which represents the product including the first intron, and a lower band (195 bp), which represents the product without the first intron. Similarly, using the primers oLX6 + 14, we showed that the major band (332 bp) contained the second intron. The positive rate of *UL111A* mRNA expression in GC tissues was 68.7% (11/16), but was only 14.3% (1/7) in the peritumoral tissue (Fig. 1h).

### High expression of UL111A in GC is associated with better prognosis

**Fig. 2:**
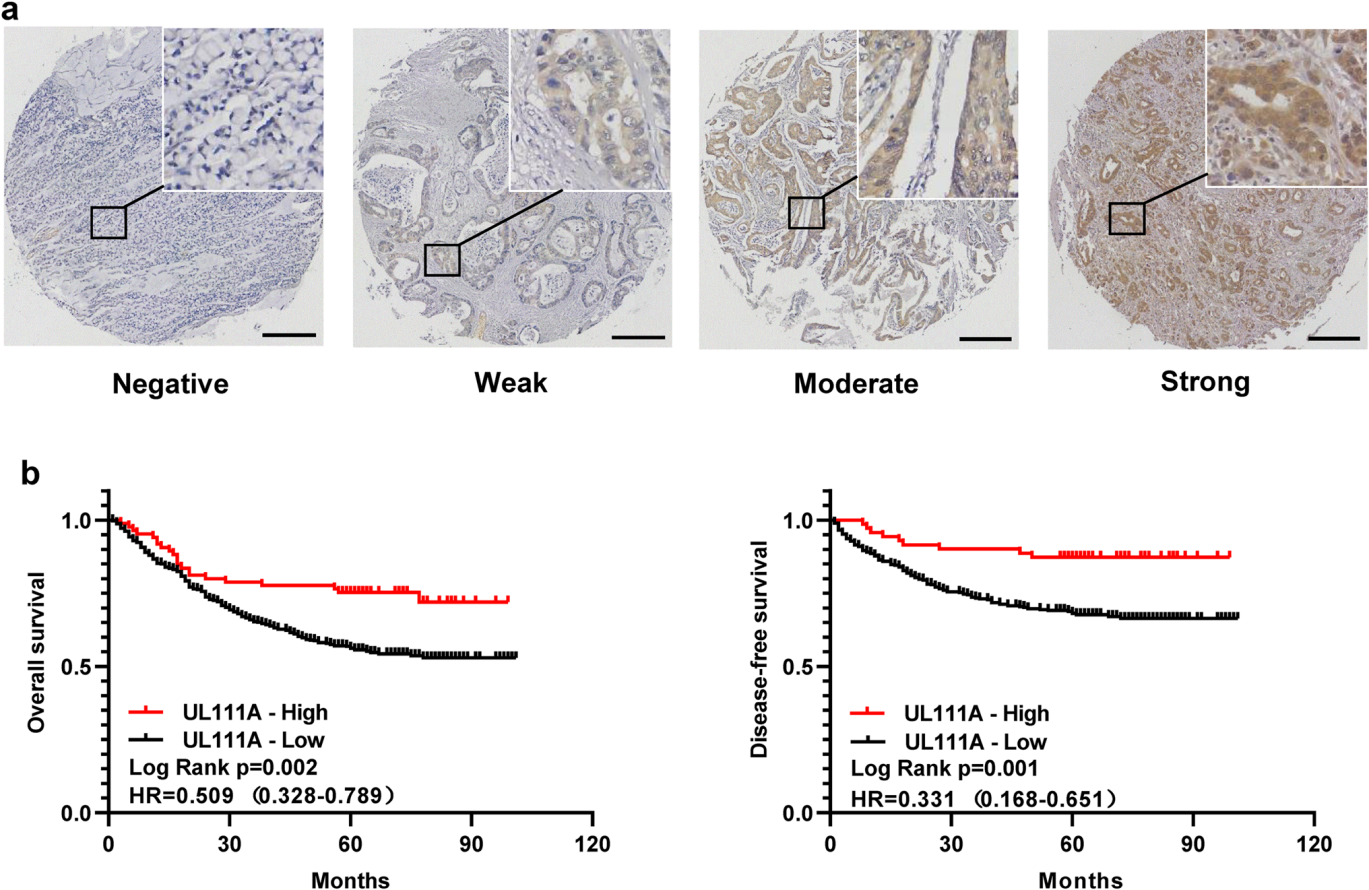
Association between UL111A expression and patient survival in GC. **a** Representative images of UL111A immunostaining in GC tissues. Bar = 300 μm. **b** High expression of UL111A predicts favorable survival in patients with GC. Overall survival (OS) and disease-free survival (DFS) are presented. Log-rank *p* values and HRs from the univariate Cox analysis are shown

To investigate the association between UL111A protein expression levels and patient survival, we performed IHC on our TMAs, which included 620 stage I–IV GC patients who underwent curative surgery and regular follow-up in the Wenzhou cohort. Figure 2a shows the different degrees of staining with GC tissues. To determine the association between UL111A expression and patient characteristics, the low and high UL111A expression groups were analyzed based on clinicopathologic parameters. Table 1 lists the characteristics of the study patients. Patients with low expression were more likely to exhibit adverse pathologic features including age < 60 (*p* = 0.040), tumor diameter ≥ 4 cm (*p* = 0.032), diffuse-type GC (*p* < 0.001), poorly differentiated and undifferentiated GC (*p* < 0.001), lymph node metastasis (*p* = 0.007), higher TNM stage (*p* = 0.032), and higher invasion depth (*p* = 0.061). However, the association with the depth of invasion was not statistically significant. Next, Kaplan–Meier analysis showed that patients with high UL111A protein expression levels had longer OS (*p* = 0.002, HR = 0.509, 95% CI = 0.328–0.789) and disease-free survival (DFS) (*p* = 0.001, HR = 0.331, 95% CI = 0.168–0.651) than patients with low UL111A protein expression levels (Fig. 2b). Moreover, survival analysis of the four subgroups, negative, weakly positive, moderately positive, and strongly positive, showed that higher UL111A protein expression was associated with better prognosis with respect to OS (supplementary Fig. 2a) and DFS (supplementary Fig. 2b). Furthermore, compared with the UL111A-negative group, the UL111A-positive group was also found to predict better OS (supplementary Fig. 2c) and DFS (supplementary Fig. 2d) in GC patients.

**Table 1:** The clinical characteristics of gastric cancer patients according to UL111A expression

Variables	Number of patients (%)		*p* value^a^
	Low expression	High expression	
Age (*n* [%])			**0.04**
< 60	277 (53.2%)	37 (41.1%)	
≥ 60	244 (46.8%)	53 (58.9%)	
Gender (*n* [%])			0.331
Male	364 (69.1%)	69 (74.2%)	
Female	163 (30.9%)	24 (25.8%)	
Adjuvant chemotherapy (*n* [%])			0.389
No	153 (29.2%)	31 (33.7%)	
Yes	371 (70.8%)	61 (66.3%)	
Serum CEA (ng/mL) (*n* [%])			1.000
< 5	400 (80.8%)	72 (80.9%)	
≥ 5	95 (19.2%)	17 (19.1%)	
Serum CA19-9 (U/mL) (*n* [%])			0.112
< 37	404 (84.5%)	65 (77.4%)	
≥ 37	74 (15.5%)	19 (22.6%)	
Serum CA72-4 (U/mL) (*n* [%])			0.751
< 6.9	311 (78.7%)	53 (76.8%)	
≥ 6.9	84 (21.3%)	16 (21.3%)	
Diameter (cm)			**0.032**
< 4	235 (44.7%)	53 (57%)	
≥ 4	291 (55.3%)	40 (43%)	
Lauren type (*n* [%])			**< 0.001**
Intestinal type	231 (43.8%)	74 (79.6%)	
Diffuse type	296 (56.2%)	19 (20.4%)	
Grade (*n*[%])			**< 0.001**
Well and moderately	168 (31.9%)	49 (52.7%)	
Poorly and undifferentiated	359 (68.1%)	44 (47.3%)	
Depth of invasion (*n* [%])			0.061
T1 + T2	182 (34.5%)	42 (45.2%)	
T3 + T4	345 (65.5%)	51 (54.8%)	
Lymph node metastasis (*n* [%])			**0.007**
No	227 (36.7%)	31 (50%)	
Yes	391 (63.3%)	31 (50%)	
TNM^b^ stage (*n* [%])			**0.032**
I	137 (26%)	36 (38.7%)	
II	110 (20.9%)	21 (22.6%)	
III	269 (51%)	36 (38.7%)	
IV	11 (2.1%)	0 (0%)	

*CEA* carcinoembryonic antigen, *CA199* carbohydrate antigen; 19-9, *CA72-4* carbohydrate antigen 72-4, *TNM* tumor, node, metastasis

^a^Values in bold are statistically significant

^b^According to AJCC/UICC Classification for Carcinoma of the Stomach (8th edition)

Further, the low UL111A expression group had significantly shorter cumulative survival than did the high UL111A expression group based on univariate analysis (Table 2). Other factors that significantly predicted OS and DFS outcomes based on univariate analysis included Lauren type, adjuvant chemotherapy, serum CEA, serum CA19-9, serum CA72-4, diameter (cm), depth of invasion, lymph node involvement, TNM stage, histologic grade, and Borrmann type (Table 2). Cox multivariate analysis showed that high UL111A protein expression was an independent predictor of OS, with an HR of 0.463 (95% CI, 0.266–0.808), and of DFS, with an HR of 0.258 (95% CI, 0.112–0.597). These details are provided in Table 2. Moreover, combined data indicated that UL111A was an independent prognostic factor for cumulative survival.

**Table 2:** Cox regression analysis of UL111A protein expression and clinicopathological covariates in patients with gastric cancer

Variables	Overall survival				Disease-free survival			
	Univariate analysis	*p* value^a^	Multivariate analysis	*p* value^a^	Univariate analysis	*p* value^a^	Multivariate analysis	*p* value^a^
	HR (95% CI)		HR (95% CI)		HR (95% CI)		HR (95% CI)	
UL111A expression (high level vs low level)	0.509 (0.328–0.789)	**0.003**	0.463 (0.266–0.808)	**0.007**	0.331 (0.168–0.651)	**0.001**	0.258 (0.112–0.597)	**0.002**
Lauren type (intestinal vs diffuse)	1.732 (1.334–2.249)	**< 0.001**			1.889 (1.336–2.670)	**< 0.001**		
Adjuvant chemotherapy (yes vs no)	2.718 (1.946–3.797)	**< 0.001**	1.547 (1.004–2.384)	**0.048**	3.202 (2.045–5.015)	**< 0.001**	1.842 (1.042–3.256)	**0.036**
Serum CEA (ng/mL) (≥ 5 vs < 5)	1.993 (1.480–2.683)	**< 0.001**			2.397 (1.636–3.511)	**< 0.001**		
Serum CA19-9 (U/mL) (≥ 37 vs < 37)	2.027 (1.466–2.804)	**< 0.001**	1.662 (1.151–2.400)	**0.007**	2.528 (1.681–3.802)	**< 0.001**	2.182 (1.395–3.415)	**0.001**
Serum CA72-4 (U/mL) (≥ 6.9 vs < 6.9)	1.896 (1.360–2.642)	**< 0.001**			1.939 (1.261–2.982)	**0.003**		
Diameter(cm) (≥ 4 vs < 4)	4.046 (2.988–5.478)	**< 0.001**	2.387 (1.579–3.608)	**< 0.001**	5.065 (3.357–7.641)	**< 0.001**	3.446 (2.045–5.808)	**< 0.001**
Depth of invasion (T3 + T4 vs T1 + T2)	6.063 (4.106–8.951)	**< 0.001**			7.807 (4.562–13.359)	**< 0.001**		
Lymph node involvement (yes vs no)	5.444 (3.782–7.838)	**< 0.001**	2.747 (1.694–4.455)	**< 0.001**	5.992 (3.725–9.637)	**< 0.001**	3.147 (1.668–5.938)	**< 0.001**
TNM stage (III + IV vs I + II)	5.081 (3.758–6.870)	**< 0.001**			5.498 (3.725–8.115)	**< 0.001**		
Histologic grade (undifferentiated + poorly vs well + moderately)	1.615 (1.217–2.144)	**0.001**	1.429 (1.004–2.033)	**0.047**	1.471 (1.024–2.111)	**0.037**		
Borrmann type (3 + 4 vs 1 + 2)	2.584 (1.935–3.452)	**0.003**	1.454 (0.965–2.192)	**0.074**	3.222 (2.171–4.781)	**< 0.001**		

*HR* hazard ratio, *CI* confidence interval

^a^A Cox regression model was used in statistical analyses. Values in bold are statistically significant

### Predictive value of UL111A protein expression for postoperative adjuvant chemotherapy

**Fig. 3:**
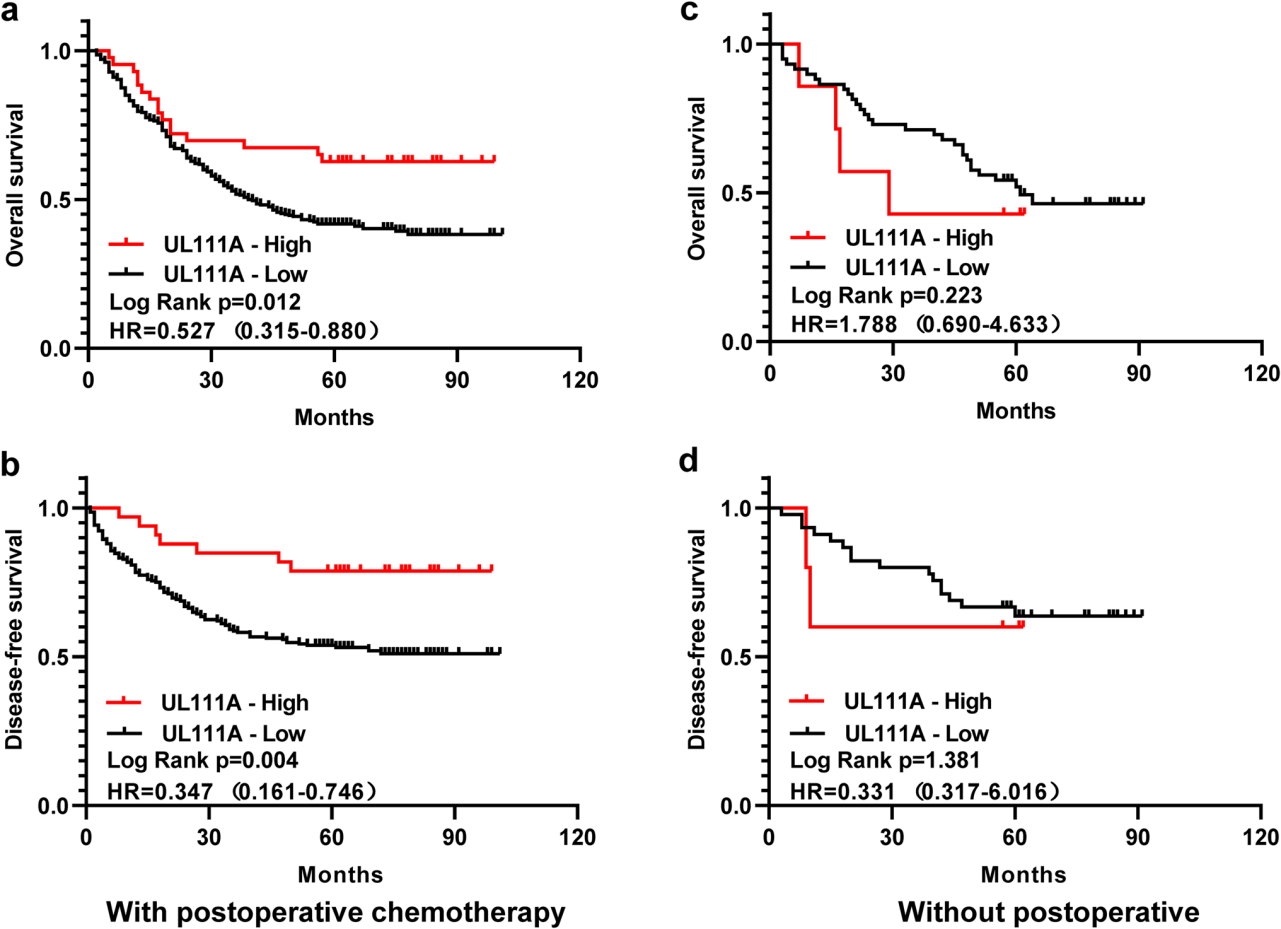
Relationship between UL111A protein expression and benefit from postoperative adjuvant chemotherapy. Association between expression of UL111A and OS (**a**) and DFS (**b**) of patients with postoperative chemotherapy. Association between expression of UL111A and OS (**c**) and DFS (**d**) of patients without postoperative chemotherapy

First, we divided the patients with TNM II/III GC into two groups, namely with or without postoperative ACT, and then analyzed whether UL111A expression can be used as a marker to predict postoperative ACT success. Interestingly, for patients who had received postoperative ACT, the high UL111A expression group was found to have longer OS (*p* = 0.012, HR = 0.527, 95% CI = 0.315–0.880) and DFS (*p* = 0.004, HR = 0.347, 95% CI = 0.161–0.746) than the low expression group (Fig. 3a, b). However, for patients who did not receive postoperative ACT, no differences in OS and DFS were observed between the two groups (Fig. 3c, d). In conclusion, we believe that UL111A protein expression might be used as a marker to predict postoperative ACT success in patients with GC.

### High UL111A expression is associated with increased T-cell infiltration

**Fig. 4:**
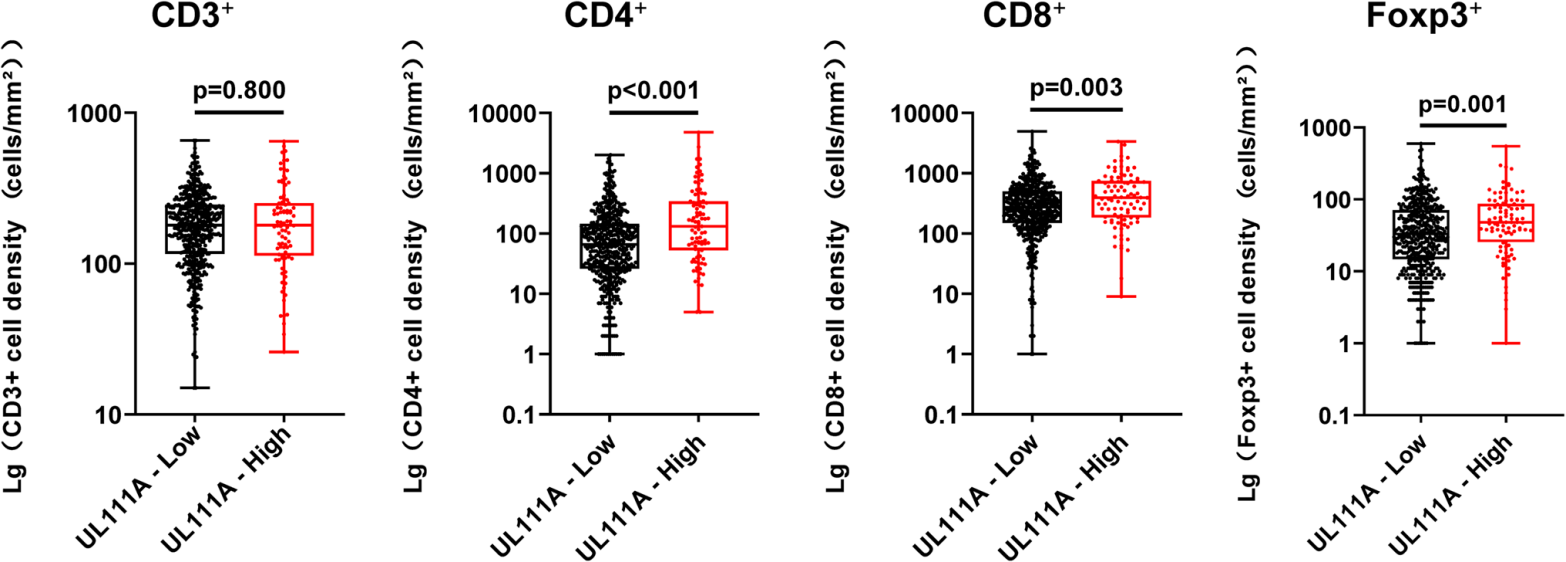
Association between various infiltrating T cells and UL111A protein expression. Infiltrating T cells grouped according to low and high expression levels of UL111A. *Lg* log 10

To investigate the relationship between different UL111A protein levels and infiltrating T cells, we also compared CD3^+^, CD4^+^, CD8^+^, and Foxp3^+^ cells in the same TMAs data and found that compared with those in the low UL111A expression group, CD4^+^, CD8^+^, and Foxp3^+^ T cells were significantly upregulated in the high UL111A expression group (CD4^+^, *p* < 0.001; CD8^+^, *p* = 0.003; Foxp3^+^, *p* = 0.001), but the association for the CD3^+^ group was not statistically significant (CD3^+^, *p* = 0.800; Fig. 4). Supplementary Fig. 3 presents respective staining of infiltrating immune cells.

### Ectopic expression of LAcmvIL-10 and cmvIL-10 inhibits the growth capacity, migration, and invasion of GC cells in vitro

**Fig. 5:**
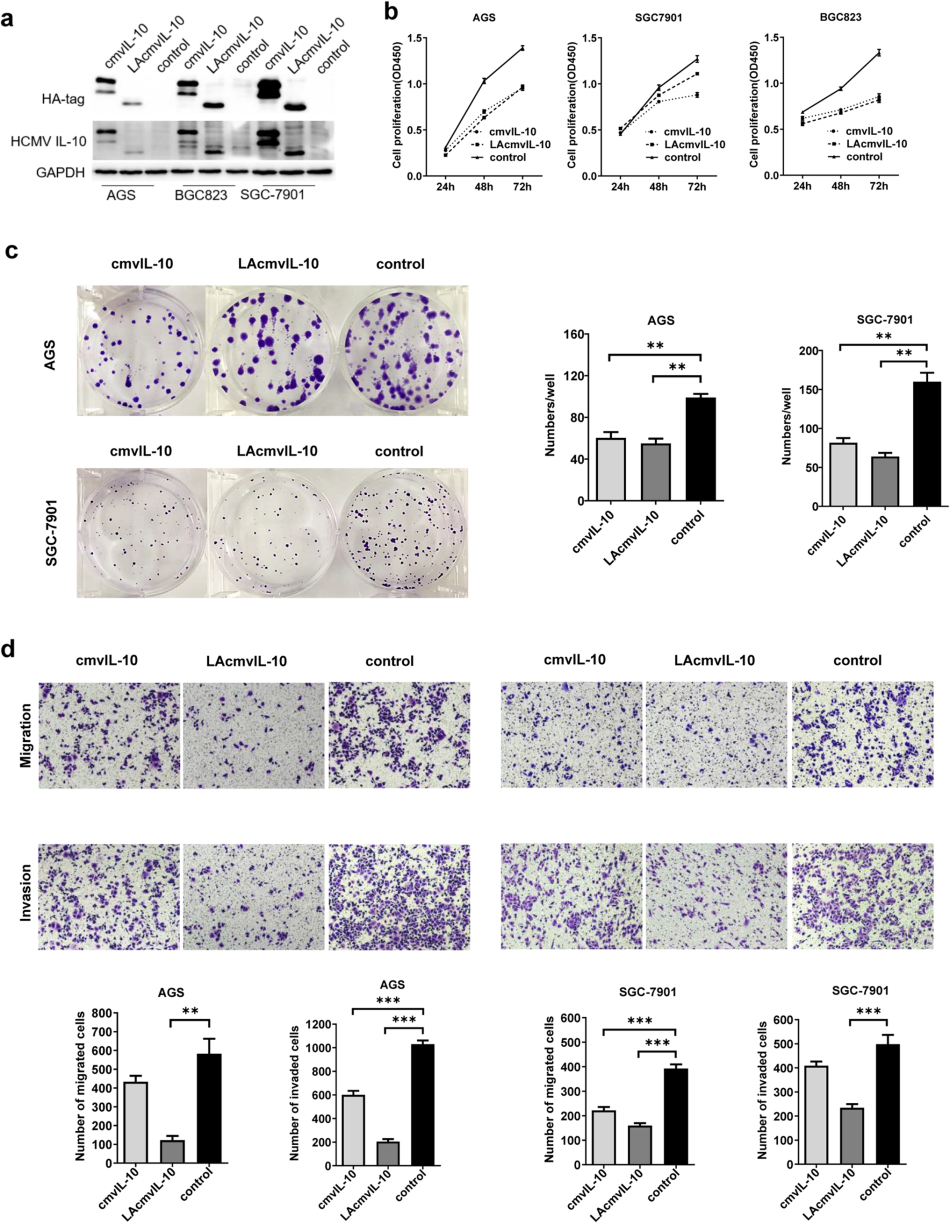
LAcmvIL-10 and cmvIL-10 suppress tumor proliferation, migration, invasion, and colony formation in vitro. **a** The expression of recombinant LAcmvIL-10 and cmvIL-10 in GC cell lines by western blotting. GAPDH was used as an internal control. The cell proliferation (**b**), colony formation (**c**), and migration and invasion (**d**) of GC cells were examined. Error bars represent the standard error of the mean obtained from three independent experiments. *p < 0.05; **p < 0.01; ***p < 0.001

To explore the effect of LAcmvIL-10 and cmvIl-10 on cell proliferation and colony formation, we utilized the GC cell lines AGC, BGC-803, and SGC-7901, and transfected them with pcDNA3.1-LAcmvIL-10-HA-tag, pcDNA3.1-cmvIL-10-HA-tag, or empty pcDNA3.1(+). The expression of LAcmvIL-10 and cmvIL-10 proteins was assessed by western blotting (Fig. 5a). CCK8 assays showed that following culture for 48 h, the proliferation ability was significantly attenuated as compared with that in the control group (Fig. 5b). Colony formation in AGS and SGC-7901 cells was also significantly decreased as compared with that in the control group (Fig. 5c). Next, to elucidate the role of UL111A in GC cell metastasis, its effects on the migration and invasion of GC cells were analyzed in vitro. Transwell assays showed that both the migratory and invasive activities of GC cells (AGS and SGC-7901) were suppressed by the expression of LAcmvIL-10 and cmvIL-10 relative to those in the control group (Fig. 5d). Therefore, our results clearly indicate that LAcmvIL-10 and cmvIL-10 inhibit GC cell growth and suppress migration and invasion in vitro.

## Discussion

In this study, we reported for the first time that UL111A is expressed in GC tissues. Previous studies have suggested that viral interleukin 10 (vIL-10) can be detected in the peripheral blood of healthy blood donors, which suggests that it might play a key role in sensing or modifying the host environment during latency (Young et al. 2017). We then uncovered that LAcmvIL-10 might be the major transcript of *UL111A* in GC tissues compared with the cmvIL-10 transcript. In addition, an unspliced isoform was detected most often in these tissues. This is the first report that shows the expression profiles of UL111A in GC tissues. Previous studies have analyzed the functions of cmvIL-10, which was first identified during lytic infection (Kotenko et al. 2000; Lockridge et al. 2000). However, there are few studies showing the biological function of LAcmvIL-10, which is expressed during latency. Studies have suggested that LAcmvIL-10 plays a role in regulating cellular miRNA expression and that this affects the levels of specific secreted cellular proteins (Fu et al. 2014).

Our IHC results also indicated that UL111A was completely distributed in the cytoplasm of GC epithelial cells and that patients with high UL111A expression had better OS and DFS than those with low UL111A expression. Remarkably, our study is the first to reveal the predictive value of UL111A with respect to postoperative ACT. Specifically, patients with high UL111A protein expression levels had superior survival after postoperative ACT.

Studies on tumor immunity have provided a wealth of information about tumor immune surveillance status and immunotherapy options (Fridman et al. 2017; Gajewski et al. 2013). Therefore, we investigated the correlation between UL111A protein levels and CD3^+^, CD4^+^, CD8^+^, and Foxp3^+^ infiltrating T-cell density (cells/mm^2^). High UL111A protein expression was positively correlated with CD4^+^, CD8^+^, and Foxp3^+^ T cells and patients with high-density infiltrating T cells had significantly longer survival than those with the corresponding low-density cells, indicating that higher UL111A protein might result in the recruitment of these infiltrating T cells. Previous evidence demonstrated that tumor-infiltrating lymphocytes, as well as Foxp3+ T cells, correlate with good prognosis in GC (Lee et al. 2008, 2018; Feichtenbeiner et al. 2013). However, the mechanism underlying this association between UL111A and immune context requires further investigation. cmvIL-10 consists of three exons that encode a protein of 175 amino acids; however, it shares only 27% amino acid homology with human IL-10 (hIL-10), so its binding capacity to IL-10R1 is stronger than that of hIL-10 (Jones et al. 2002). Some studies have shown that IL-10 plays a role in immunosuppression (Saraiva and O’Garra 2010; Ouyang et al. 2011). Similar to hIL-10, cmvIL-10 also has several immunosuppressive properties including the ability to decrease major histocompatibility complex (MHC) class I and MHC class II expression on monocytes, inhibit the production of pro-inflammatory cytokines by lipopolysaccharide-stimulated peripheral blood mononuclear cells (PBMCs), monocytes, and monocyte-derived DCs through the IL-10 receptor, decrease matrix metalloproteinase activity in endothelial cells and cytotrophoblasts, and impair endothelial cell migration and cytotrophoblast invasiveness in vitro (Slobedman et al. 2009; Spencer et al. 2002; Spencer 2006; Raftery et al. 2004; Yamamoto-Tabata et al. 2004). In addition, through surface IL-10R expression in MDA-MB-231 and MCF-7 breast cancer cells, cmvIL-10 protein was found to promote their proliferation, migration, and metastatic potential (Valle Oseguera and Spencer 2014; Bishop et al. 2015). LAcmvIL-10, alternatively spliced from the viral *UL111A* gene, consists of 139 amino acids, has a C-terminal truncation, and can be identified during both latent and productive infection (Jenkins et al. 2004; Jenkins et al. 2008a, b). Similarly, like cmvIl-10, LAcmvIL-10 can also suppress MHC class II expression by primary human myeloid progenitor cells and monocytes, but the mechanism might differ from that of cmvIL-10 (Jenkins et al. 2008a, b; Poole et al. 2014; Cheung et al. 2009). Unlike cmvIL-10, LAcmvIL-10 does not trigger the phosphorylation of Stat3, and its ability to downregulate MHC-II was not blocked by neutralizing antibodies to hIL-10R, suggesting that LAcmvIL-10 either does not engage cellular IL-10R or that it utilizes it in a manner different from that utilized by cmvIL-10 (Jenkins et al. 2008a, b).

The modulation of secreted cellular proteins by HCMV during latent infection and productive infection has been reported. During latency, LAcmvIL-10 modulates cellular IL-10 and CCL8 secretion through changes in the cellular microRNA hsa-miR-92a (Poole et al. 2014). The authors also showed that human cytomegalovirus latency alters the cellular secretome, which can first result in the recruitment of CD4^+^ T cells and then the inhibition of their antiviral effector functions, thereby aiding the maintenance of latent infection in the face of the host immune response (Mason et al. 2012). As is known, both CD4^+^ and CD8^+^ T cells play critical roles in the cell-mediated control of HCMV infection and clinical disease (Sylwester et al. 2005). Previous studies also suggested that latency-associated UL111A could promote MHC class II downregulation and inhibit CD4 + T cell activation (Cheung et al. 2009). Using a *UL111A*-deletion virus, one study found that the role of this gene is to downregulate the CD4^+^ T cell recognition of latently infected cells, which probably enhances the capacity of HCMV to persist in a latent state within the human host (Cheung et al. 2009). Therefore, we considered that this mechanism might also exist in GC patients, which needs to be further explored. Still, our data demonstrated that the UL111A, which encodes cmvIL-10 and LAcmvIL-10, was shown to effectively inhibit the malignancy of GC cells. Recently, one report suggested that cmvIL-10 rapidly and independently augments NK cell cytotoxicity through most activating receptors, which may benefit HCMV persistence or contribute to NK cell function against viral infections and cancers (Holder and Grant 2019). Therefore, we suppose that the function of UL111A on GC may be due to innate immune NK cells that play a part of the function, which requires further in vivo experiments to determine.

Next, we further detected *UL111A* transcripts in GC cell lines infected by the AD169 and Merlin virus (supplementary Fig. 4). As previously reported in MCF-7 and MDA-MB-231 breast cancer cell lines, cmvIL-10 protein showed a tumorigenic effect (Valle Oseguera and Spencer 2014; Bishop et al. 2015). Other researchers also found that cmvIL-10 protein could enhance the migration of glioma cancer stem cells (Dziurzynski et al. 2011). However, we found that exogenous cmvIL-10 had no significant effect on GC cell lines (supplementary Fig. 5), whereas the overexpression of LAcmvIL-10 and cmvIL-10 significantly inhibited the growth and malignancy of GC cells. The mechanism underlying this association between LAcmvIL-10 or cmvIL-10 and such anti-tumor effects still needs further investigation. Our study also has some limitations. First, the number of samples for detecting UL111A transcripts in GC tissues is too few and there may be other undetected transcripts. Second, considering the mechanism of tumor immunity, the correlation between the expression of markers such as PD-L1, PD-1 or CTLA-4 and the expression level of UL111A protein may require further study. Third, we carried out the present study at the in vitro level. However, the oncological properties of UL111A-deletion virus on GC cells and experiments at the animal level remain to be further explored and verified.

## Conclusions

In summary, our study showed that high UL111A expression changes T-cell infiltration and is associated with better patient survival. Most importantly, high UL111A expression was found to be an independent prognostic factor of cumulative survival and might predict superior response to postoperative ACT in patients with GC. However, further prospective studies as well as more detailed mechanistic studies are needed.

## Electronic supplementary material

Below is the link to the electronic supplementary material.

Supplementary file1 (DOCX 6873 kb)

## Abbreviations

HCMV: Human cytomegalovirus
GC: Gastric cancer
ACT: Adjuvant chemotherapy
LAcmvIL-10: Latency-associated viral interleukin-10
OS: Overall survival
GMPs: Granulocyte–macrophage progenitors
HFFs: Human foreskin fibroblasts
IHC: Immunohistochemistry
TMA: Tissue microarray
HR: Hazard ratio
CI: Confidence intervals
DFS: Disease-free survival
vIL-10: Viral interleukin 10
hIL-10: Human IL-10
MHC: Major histocompatibility complex
PBMCs: Peripheral blood mononuclear cells
