# Supplementary material for: Overexpression of the human cytomegalovirus UL111A is correlated with favorable survival of patients with gastric cancer and changes T-cell infiltration and suppresses carcinogenesis
Source: J Cancer Res Clin Oncol. 2020 Feb 5;146(3):555–68. doi: 10.1007/s00432-019-03092-x (PMC7039847; doi:10.1007/s00432-019-03092-x)
Supplement: Supplementary file 1 — Supplementary file1 (DOCX 6873 kb) [file 432_2019_3092_MOESM1_ESM.docx]

**Overexpression of the human cytomegalovirus UL111A is correlated with favorable survival of patients with gastric cancer and changes T cell infiltration and suppresses carcinogenesis**

Xin Liu^1,2*^, Kangming Lin^2*^, Xielin Huang^1^, Wangkai Xie^1,2^, Dan Xiang^2^, Ning Ding^2^, Changyuan Hu^3^, Xian Shen^1#^, Xiangyang Xue^2#^, Yingpeng Huang^1#^

^1^Department of General Surgery, The Second Affiliated Hospital and Yuying Children’s Hospital of Wenzhou Medical University, Wenzhou, China

^2^Department of Microbiology and Immunology, Institute of Molecular Virology and Immunology, Institute of Tropical Medicine, School of Basic Medical Sciences, Wenzhou Medical University, Wenzhou, China

^3^ Department of Gastrointestinal Surgery, The First Afﬁliated Hospital, Wenzhou Medical University, Wenzhou, China

^*^ These authors contributed equally to this work

**Address correspondence to:** Dr. Xian Shen and Yingpeng Huang, Department of General Surgery, The Second Affiliated Hospital and Yuying Children’s Hospital of Wenzhou Medical University, Wenzhou 325035, China. Tel: +86 13968888872, E-mail: 13968888872@163.com (XS); Tel: +86 13587616686, E-mail: 171121477@qq.com (YPH) ; Dr. Xiangyang Xue, Department of Microbiology and Immunology, Institute of Molecular Virology and Immunology, Institute of Tropical Medicine, School of Basic Medical Sciences, Wenzhou Medical University, Wenzhou 325006, China. Tel: +86 15058788169 E-mail: wzxxy001@163.com;

| **Table 1. The proportion of different vIL-10 subtypes in the clone sequence** | | | |
| --- | --- | --- | --- |
| Isoforms | Percentage of clones | | |
|  | Sample 1 | Sample 2 | Total |
| CmvIL-10 | 0/28 | 1/25 | 1/53 |
| LAcmvIL-10 | 4/28 | 3/25 | 7/53 |
| Unspliced | 24/28 | 21/25 | 45/53 |

| **Table 2. UL111A oligo primers used in this study** | | |  |
| --- | --- | --- | --- |
|  |  |  |  |
| **Primer#** | **Oligo#** | **Sequence (5'-3')** | **Orientation** |
| oLX-1 | vIL-10AFP | ATGCTGTCGGTGATGGTC | Forward |
| oLX-2 | vIL-10CFP | CGACGATAAAGAATACAAAGCC | Forward |
| oLX-3 | vIL-10EFP | AAAGCCGCAGTGTCGTCCAG | Forward |
| oLX-4 | vIL-10FFP | GTCCGCACAGAGGTAACAA | Forward |
| oLX-5 | vIL-10GFP | GTGATGGTCTCTTCCTCT | Forward |
| oLX-6 | vIL-10IFP | ACGTGAGGACGACTACTCCG | Forward |
| oLX-7 | vIL-10JFP | GTTTTCCTCTTGTAGCAACGTG | Forward |
| oLX-8 | vIL-10HFP | CATCATAACATAAAGGACCACC | Forward |
| oLX-9 | vIL-10KFP | CGATAACCCGTCAGATTGA | Forward |
| oLX-10 | BvIL-10FP | CGCGGATCCGCCACCATGCTGTCGGTGATGGTC | Forward |
| oLX-19 | GAPDH-FP | TGAACGGGAAGCTCACTGG | Forward |
| oLX-11 | vIL-10ARP | CTTTCTCGAGTGCAGAT | Backward |
| oLX-12 | vIL-10CRP | CCAGATACCGCCTCAACAA | Backward |
| oLX-13 | vIL-10ERP | CATTGCCGCATGTCTTTG | Backward |
| oLX-14 | vIL-10FRP | TCCTGAGACAGCCGACTAATC | Backward |
| oLX-15 | vIL-10GRP | GTGCAGATACTCTTCGAGA | Backward |
| oLX-16 | vIL-10HRP | CTAGCAACACCCACAAACA | Backward |
| oLX-17 | vIL-10KRP | CTAGCAACACCCACAAACA | Backward |
| oLX-18a | EvIL-10RP | CCGGAATTCTTATCAAGCGTAGTCTGGGACGTCGTATGGGTACTTTCTCGAGTGCAGAT | Backward |
| oLX-18b | ELA-10RP | CCGGAATTCTTATCAAGCGTAGTCTGGGACGTCGTATGGGTACCTCTGCGCGGACA | Backward |
| oLX-20 | GAPDH-RP | TCCACCACCCTGTTGCTGTA | Backward |


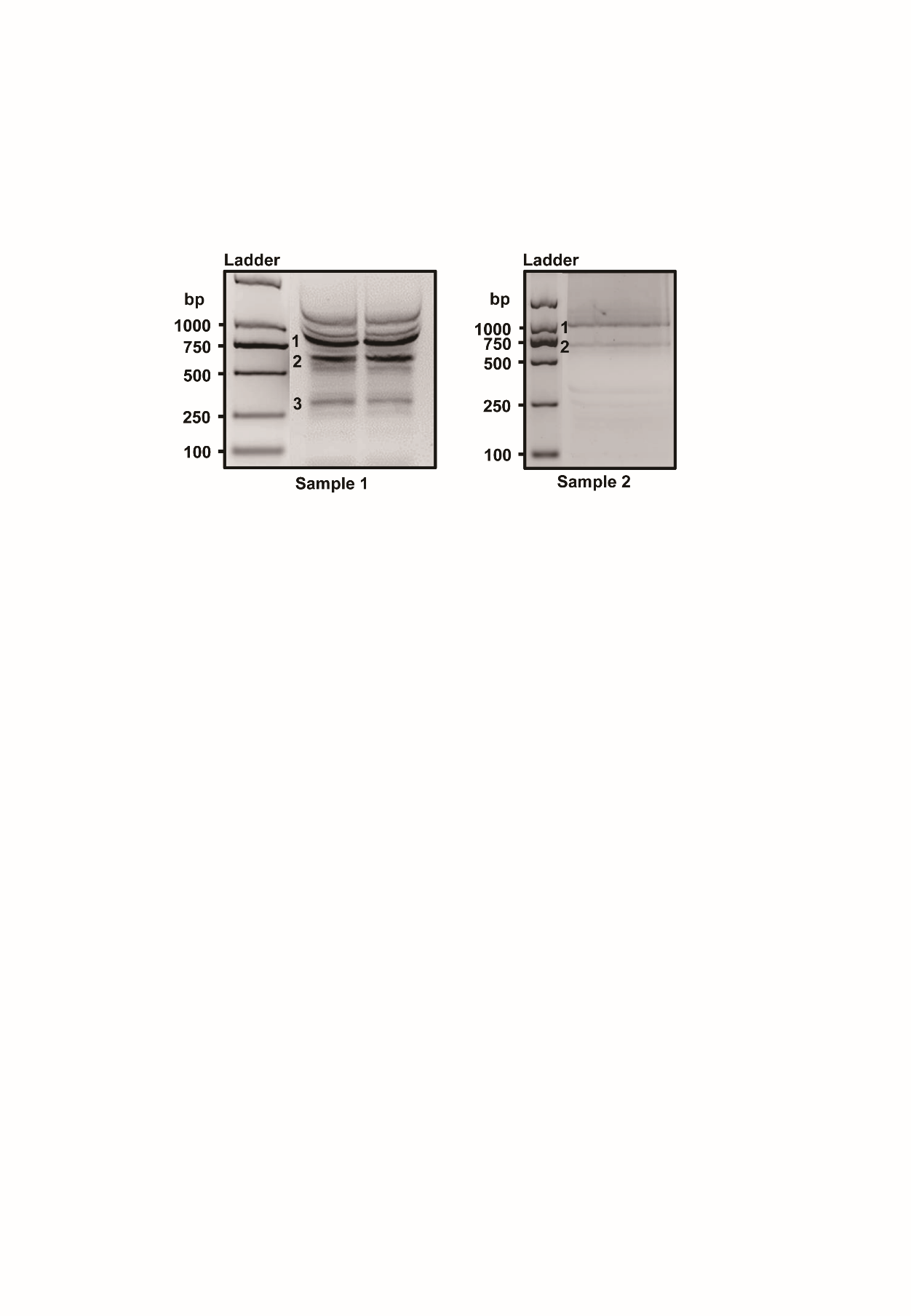


**supplementary Fig. 1. Detection of UL111A transcripts in GC tissues.** RT-PCR map of UL111A transcripts obtained using UL111A-specific full-length primers on total RNA isolated from HCMV-infected GC tissues. The bands labeled with numbers were gel-purified, cloned, and sequenced. The products of 1 and 3 were also sequenced, but not the UL111A sequence.


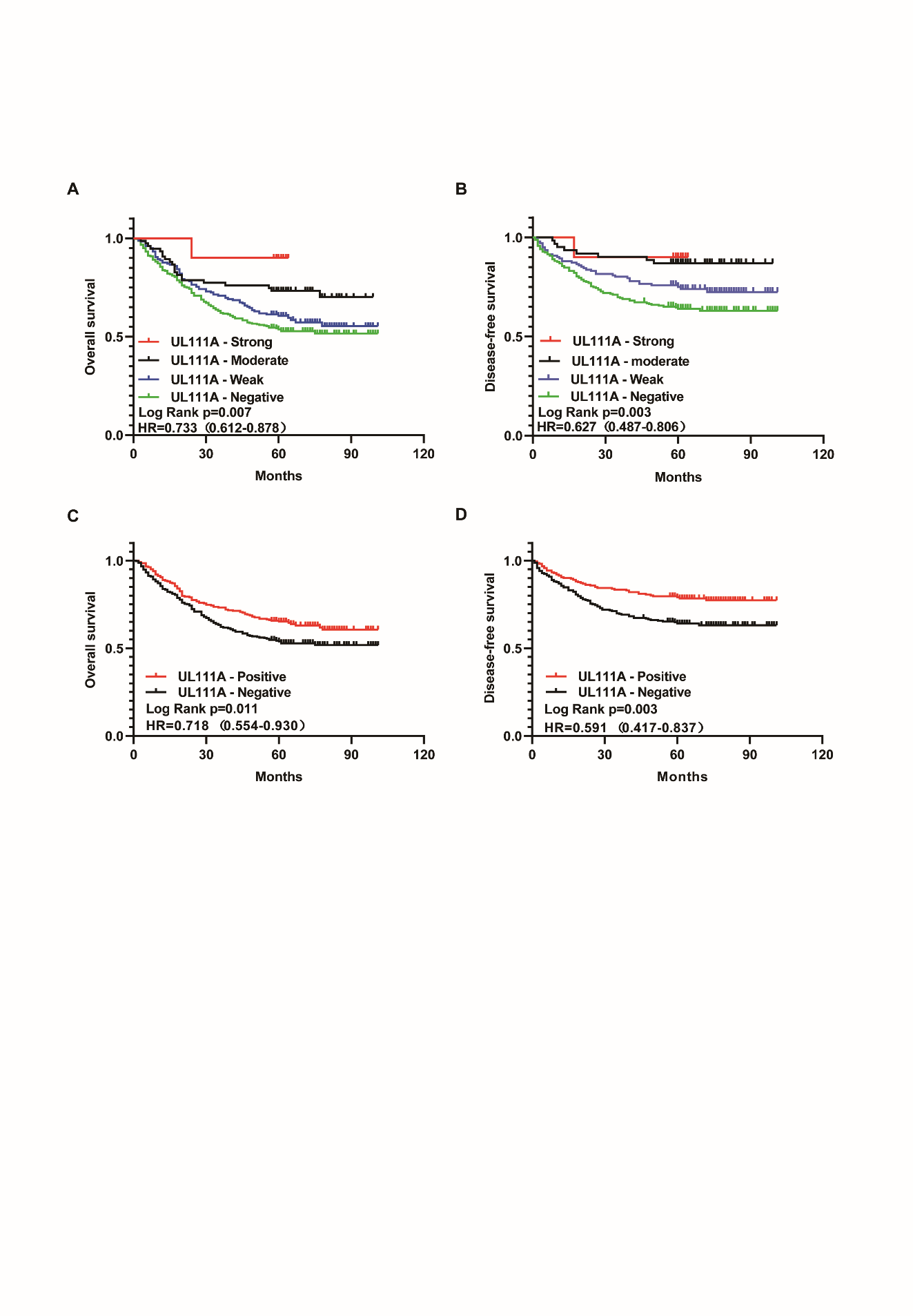


**supplementary Fig. 2. Kaplan–Meier survival analysis based on subgroups of different expression levels of UL111A protein.** Survival analysis of the four subgroups (negative, weakly positive, moderately positive, and strongly positive) showed correlation between higher UL111A levels and better prognosis in terms of OS **(A)** and DFS **(B)**. Compared to the UL111A-negative expression subgroup, UL111A positive-expression subgroup predicted a better OS **(C)** and DFS **(D)** in GC patients.


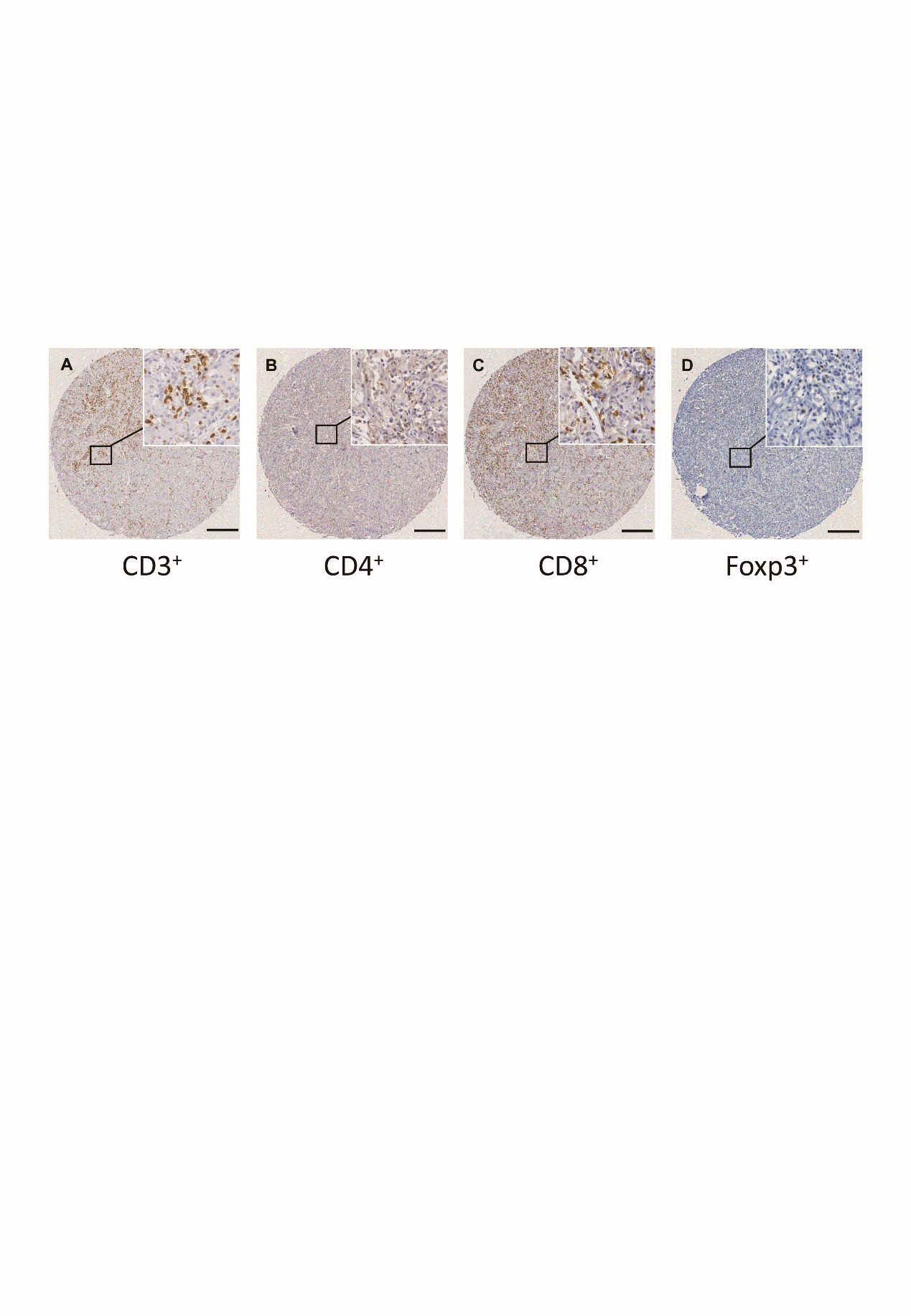


**supplementary Fig. 3. Immunohistochemical staining of CD3+, CD4+, CD8+, and Foxp3+ T cells. (A-D)** Representative images for immunostaining of CD3^+^ **(A)**, CD4^+^ **(B)**, CD8^+^ **(C)**, Foxp3^+^T **(D)** cells in GC tissues. Bar = 300um.


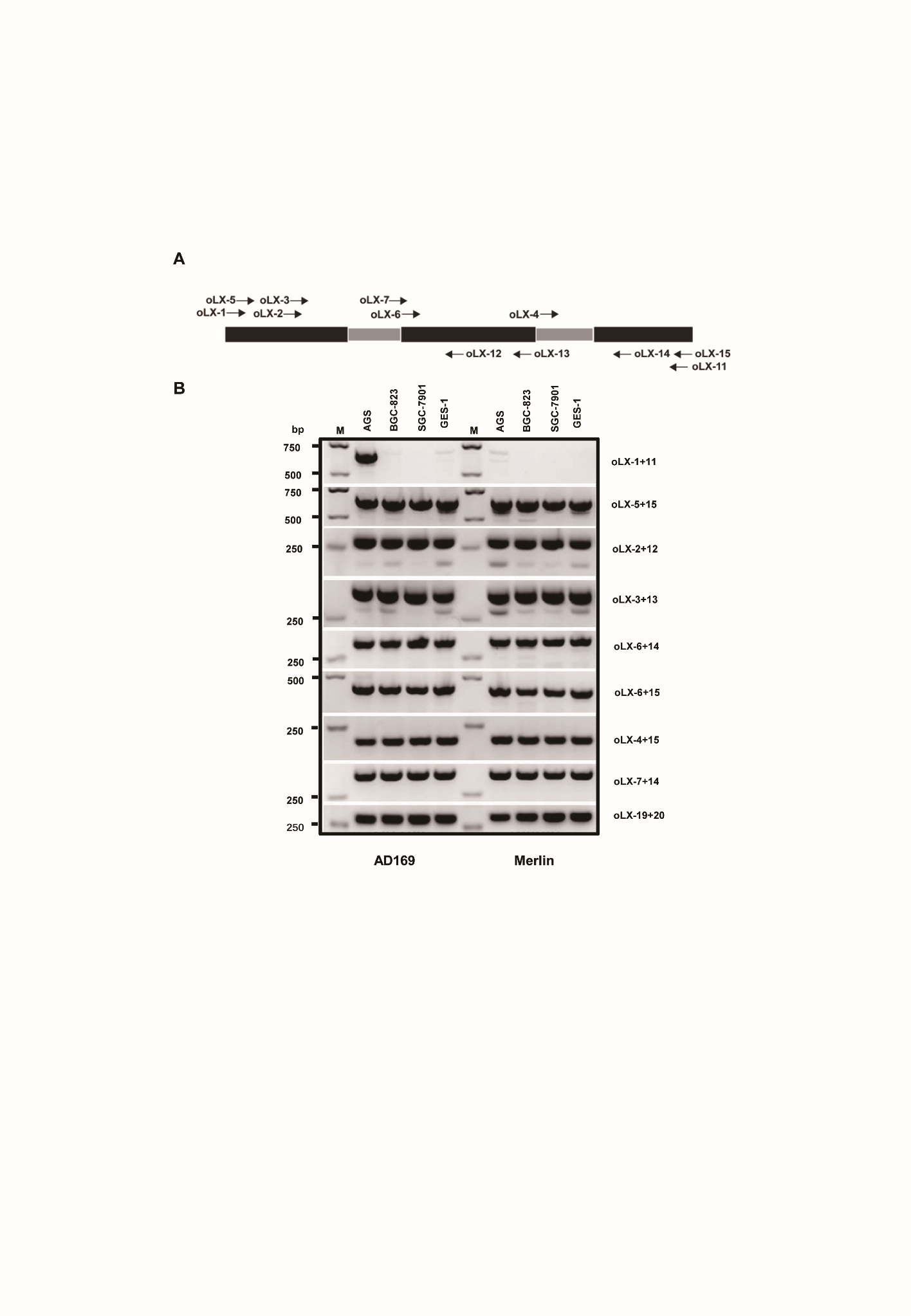


**supplementary Fig. 4. Detection of UL111A transcripts in gastric cancer cell lines. (**A). Location of different primers on the UL111A sequence used for RT-nested PCR analysis. (B). RT-nested PCR detection of intron retention and abundance of unspliced UL111A on total RNA isolated from GC cell lines and human gastric epithelium (GES-1) cells infected with AD169 and Merlin virus.

c
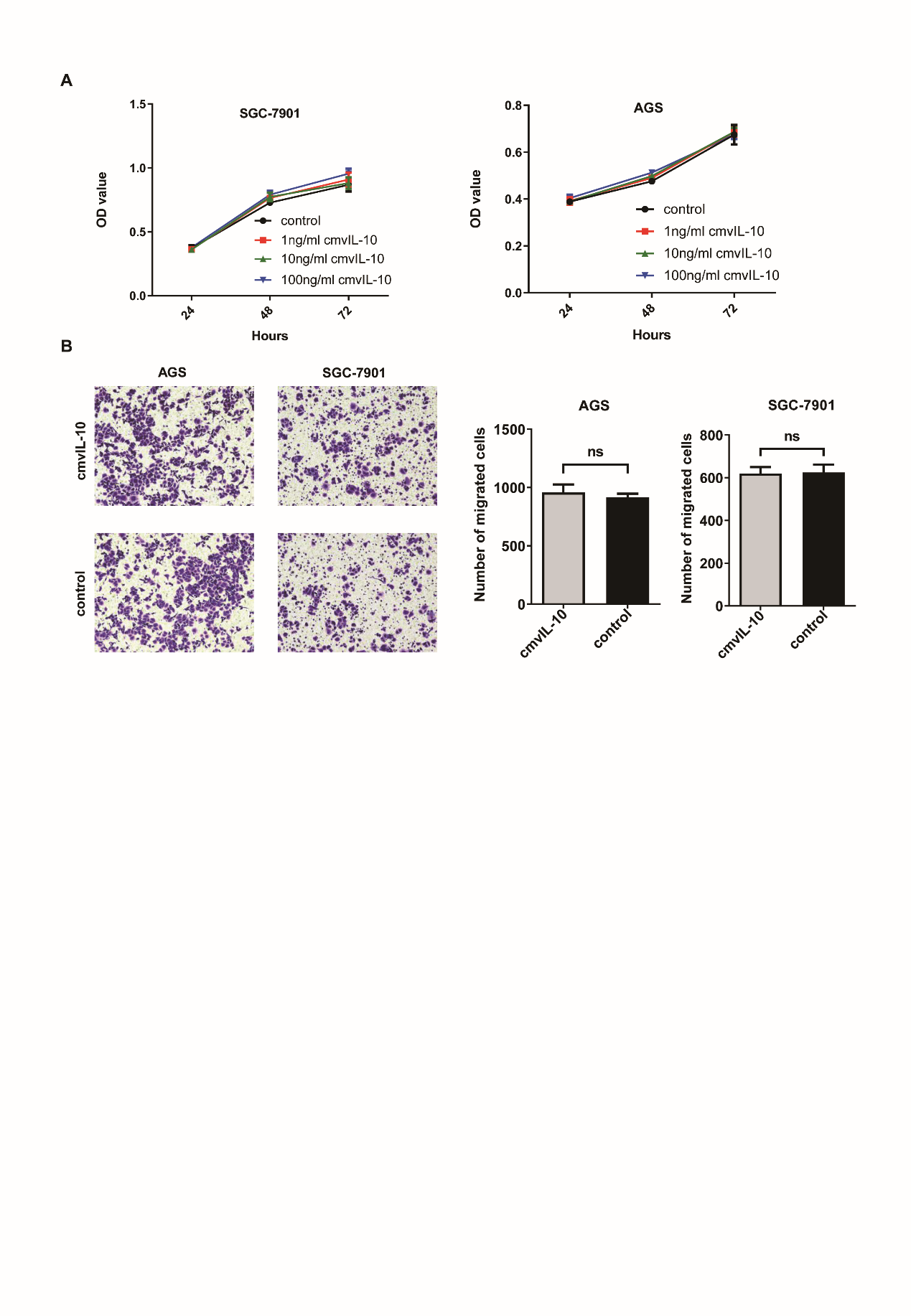


**supplementary Fig. 5.** **Effect of exogenous cmvIL-10 protein on the proliferation and migration of GC cells.** There was no statistical difference in proliferation **(A)** and migration **(B)** of AGS and SGC-7901 cells between the two groups.
